# Left Ventricular Hypertrabeculation Is Not Associated With Cardiovascular Morbity or Mortality: Insights From the Eurocmr Registry

**DOI:** 10.3389/fcvm.2020.00158

**Published:** 2020-09-22

**Authors:** Filip Zemrak, Zahra Raisi-Estabragh, Mohammed Y. Khanji, Saidi A. Mohiddin, Oliver Bruder, Anja Wagner, Massimo Lombardi, Juerg Schwitter, Albert C. van Rossum, Günter Pilz, Detlev Nothnagel, Henning Steen, Eike Nagel, Sanjay K. Prasad, Christina C. Deluigi, Thorsten Dill, Herbert Frank, Steffen Schneider, Heiko Mahrholdt, Steffen E. Petersen

**Affiliations:** ^1^William Harvey Research Institute, NIHR Barts Biomedical Research Centre, Queen Mary University of London, London, United Kingdom; ^2^Barts Heart Centre, St Bartholomew's Hospital, Barts Health NHS Trust, London, United Kingdom; ^3^Department of Cardiology and Angiology, Elisabeth-Krankenhaus Essen, Ruhr University Bochum, Bochum, Germany; ^4^Department of Cardiology, St. Vincent's Medical Centre, Bridgeport, CT, United States; ^5^I.R.C.C.S. Multimodality Cardiac Imaging, Policlinico San Donato, Milan, Italy; ^6^Cardiac MR Centre, University Hospital (CHUV), Switzerland and Lausanne University, Lausanne, Switzerland; ^7^Department of Cardiology, Amsterdam University Centres, Amsterdam, Netherlands; ^8^Department of Cardiology, Clinic Agatharied, University of Munich, Munich, Germany; ^9^Department of Cardiology, Klinikum Ludwigsburg, Ludwigsburg, Germany; ^10^Medneo Group, Berlin, Germany; ^11^Institute for Experimental and Translational Cardiovascular Imaging DZHK (German Centre for Cardiovascular Research) Centre for Cardiovascular Imaging, Partner Site RheinMain, University Hospital, Goethe University, Frankfurt, Germany; ^12^CMR Unit, Royal Brompton Hospital, London, United Kingdom; ^13^National Heart and Lung Institute, London, United Kingdom; ^14^Department of Cardiology, Inselspital, University of Bern, Bern, Switzerland; ^15^Department of Internal Medicine, Krankenhaus Benrath, Düsseldorf, Germany; ^16^Department of Internal Medicine, University Hospital Tulln, Tulln, Austria; ^17^Institut für Herzinfarktforschung, Ludwigshafen, Germany; ^18^Department of Cardiology, Robert Bosch Medical Centre, Stuttgart, Germany

**Keywords:** left ventricular non-compaction, left ventricular trabeculation, cardiomyopathy, cardiac magnetic resonance, mortality

## Abstract

**Aim:** Left ventricular non-compaction (LVNC) is perceived as a rare high-risk cardiomyopathy characterized by excess left ventricular (LV) trabeculation. However, there is increasing evidence contesting the clinical significance of LV hyper-trabeculation and the existence of LVNC as a distinct cardiomyopathy. The aim of this study is to assess the association of LV trabeculation extent with cardiovascular morbidity and all-cause mortality in patients undergoing clinical cardiac magnetic resonance (CMR) scans across 57 European centers from the EuroCMR registry.

**Methods and Results:** We studied 822 randomly selected cases from the EuroCMR registry. Image acquisition was according to international guidelines. We manually segmented images for LV chamber quantification and measurement of LV trabeculation (as per Petersen criteria). We report the association between LV trabeculation extent and important cardiovascular morbidities (stroke, atrial fibrillation, heart failure) and all-cause mortality prospectively recorded over 404 ± 82 days of follow-up. Maximal non-compaction to compaction ratio (NC/C) was mean (standard deviation) 1.81 ± 0.67, from these, 17% were above the threshold for hyper-trabeculation (NC/C > 2.3). LV trabeculation extent was not associated with increased risk of the defined outcomes (morbidities, mortality, LV CMR indices) in the whole cohort, or in sub-analyses of individuals without ischaemic heart disease, or those with NC/C > 2.3.

**Conclusion:** Among 882 patients undergoing clinical CMR, excess LV trabeculation was not associated with a range of important cardiovascular morbidities or all-cause mortality over ~12 months of prospective follow-up. These findings suggest that LV hyper-trabeculation alone is not an indicator for worse cardiovascular prognosis.

## Introduction

Left ventricular non-compaction cardiomyopathy (LVNC) is perceived as a rare genetic cardiomyopathy characterized by abnormal arrest of *in-utero* myocardial compaction (1). Tertiary center cohorts of LVNC report association with life-threatening arrhythmias, thromboembolism, and left ventricular (LV) dysfunction (2–5). LVNC is recognized as a “genetic cardiomyopathy” by the American Heart Association and as an “unclassified cardiomyopathy” by the European Society of Cardiology (6, 7). Identification of excess LV trabeculations alongside a thin layer of compacted myocardium on non-invasive imaging is key to diagnosis. The Petersen cardiac magnetic resonance (CMR) criteria are widely used for quantification of LV trabeculations and to guide diagnosis of LVNC (8).

Increased awareness of LVNC and improved imaging techniques have led to a surge in its diagnosis. However, studies of healthy cohorts have identified fulfillment of the LVNC imaging criteria in a high proportion of individuals with no clear association to adverse outcomes (9–11). Similar findings have been reported in small studies of asymptomatic athletes and healthy pregnant women (12–14). These findings have been replicated in single center studies of symptomatic individuals and those with known structural heart disease (15, 16). Further, a multicenter study of individuals diagnosed with LVNC reports better than expected outcomes with no prognostic impact of LV trabeculation beyond known parameters such as left ventricular ejection fraction (LVEF) (17).

There is increasing uncertainty regarding the clinical significance of LV hyper-trabeculation and some have questioned the status of LVNC as a distinct cardiomyopathy (18). However, many of these studies have been conducted in low-risk populations, in whom application of diagnostic criteria is misleading.

There remains some uncertainty regarding generalisability of findings from these low-risk asymptomatic cohorts to patients seen in clinical practice. It is important to ascertain the significance of LV trabeculation in a real-life population with clinical indication for CMR. We present the first prospective multicenter multinational study of the relationship of the extent of LV trabeculation with cardiovascular morbidity and all-cause mortality in real-life patients undergoing clinical CMR in 57 European center.

## Methods

### The EuroCMR Registry

The EuroCMR registry was set up to assess the clinical utility and prognostic impact of CMR in real-life clinical scenarios. Over 37,000 consecutive patients undergoing routine clinical CMR were recruited from 57 center in 15 European countries (19). Scans were performed in compliance with standardized protocols. The only exclusion criterion was contraindication to CMR. There was prospective follow-up of patients with suspected coronary artery disease (CAD) or hypertrophic cardiomyopathy (HCM) through standardized telephone interviews (Supplementary Table 1). If contact with the patient was unsuccessful, government registration offices were contacted to obtain updated contact information or details of cause of death. The EuroCMR registry study design and protocols are detailed elsewhere (20). All participating center had approval from local institutional ethics review boards, and all patients provided written informed consent in accordance to the Declaration of Helsinki.

### Selection of CMR Studies

**Figure 1:**
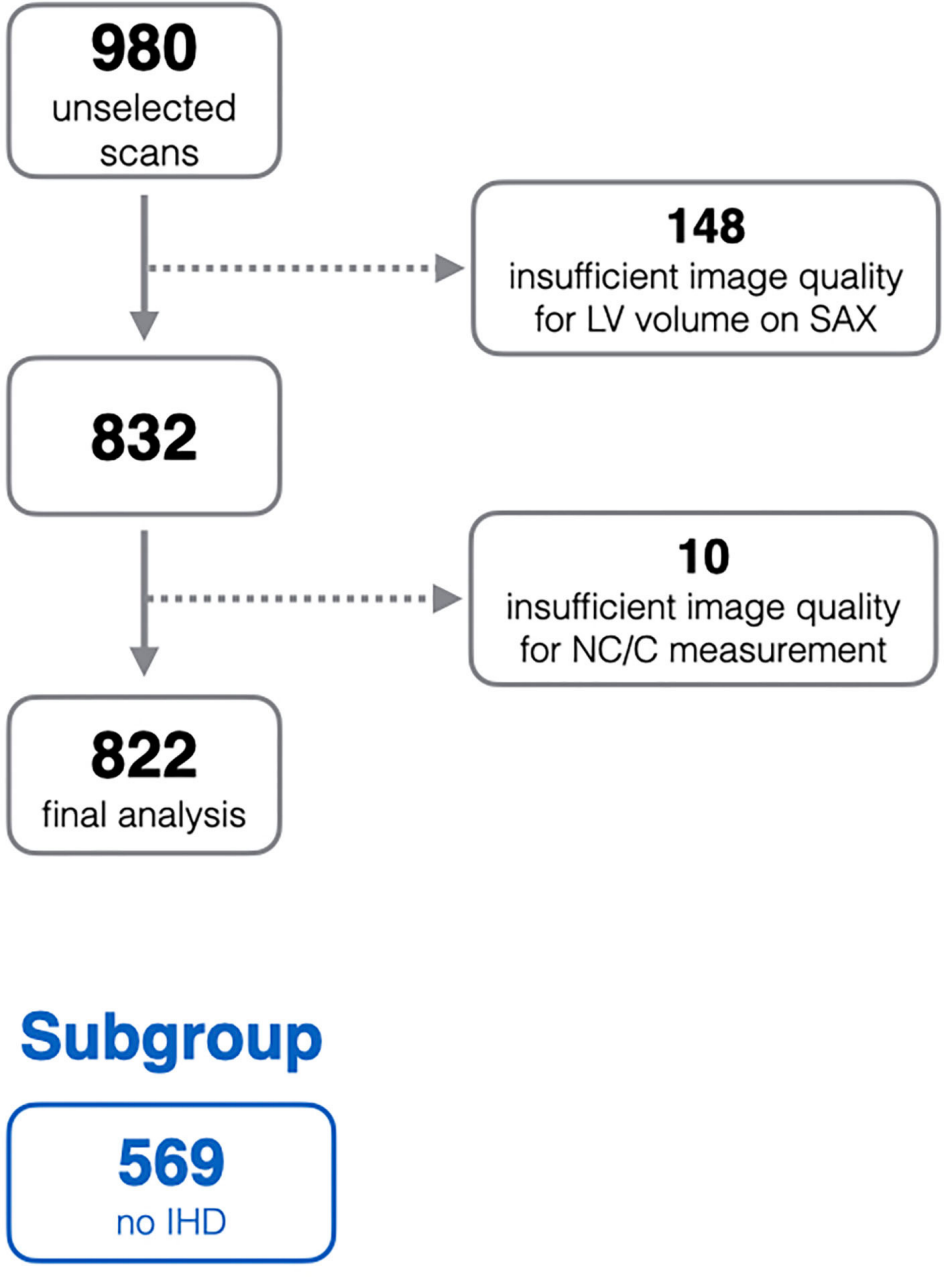
Flow diagram of exclusion process from the initial unselected scans. IHD, ischaemic heart disease; LV, left ventricle; NC/C, non-compacted to compacted ratio; SAX, short-axis stack.

In order to ensure quality control within the EuroCMR registry, there was requirement for each center to submit a selection of random cases for quality assessment. This created a bank of 980 randomly selected scans collated from all participating center. The current study is based on analysis of this sample. All scans were anonymised, and the demographic and clinical data were not available at the time of image analysis. After quality control checks, 158 scans were excluded due to sub-optimal image quality, the remaining 822 scans are included in this analysis (Figure 1).

### Measurement of LV Volumes and Function

CMR studies were analyzed using cvi42 software (Circle Cardiovascular Imaging Inc, Calgary, Canada). Left ventricular end diastolic volume (LVEDV), end systolic volume (LVESV), and end-diastolic mass (LVM) were obtained from cine short-axis images covering the LV from base to apex. End-systole and end-diastole were defined by the cardiac phases with the largest and smallest blood pool area at the mid-ventricular level. The operator defined the slice range (base to apex). Endocardial and epicardial contours were manually drawn at end-diastole and end-systole for each slice according to expert consensus recommendations (21). Papillary muscles were included in the blood pool. The cvi42 software computed LV volumes by slice summation, LVEF was calculated in the usual way. LVM was calculated by subtracting LVESV from the epicardial volume in end-diastole and multiplying it by the myocardial muscle density of 1.05 g/ml. LV parameters were indexed to body surface area (BSA, denoted by i).

### Measurement of LV Trabeculations

**Figure 2:**
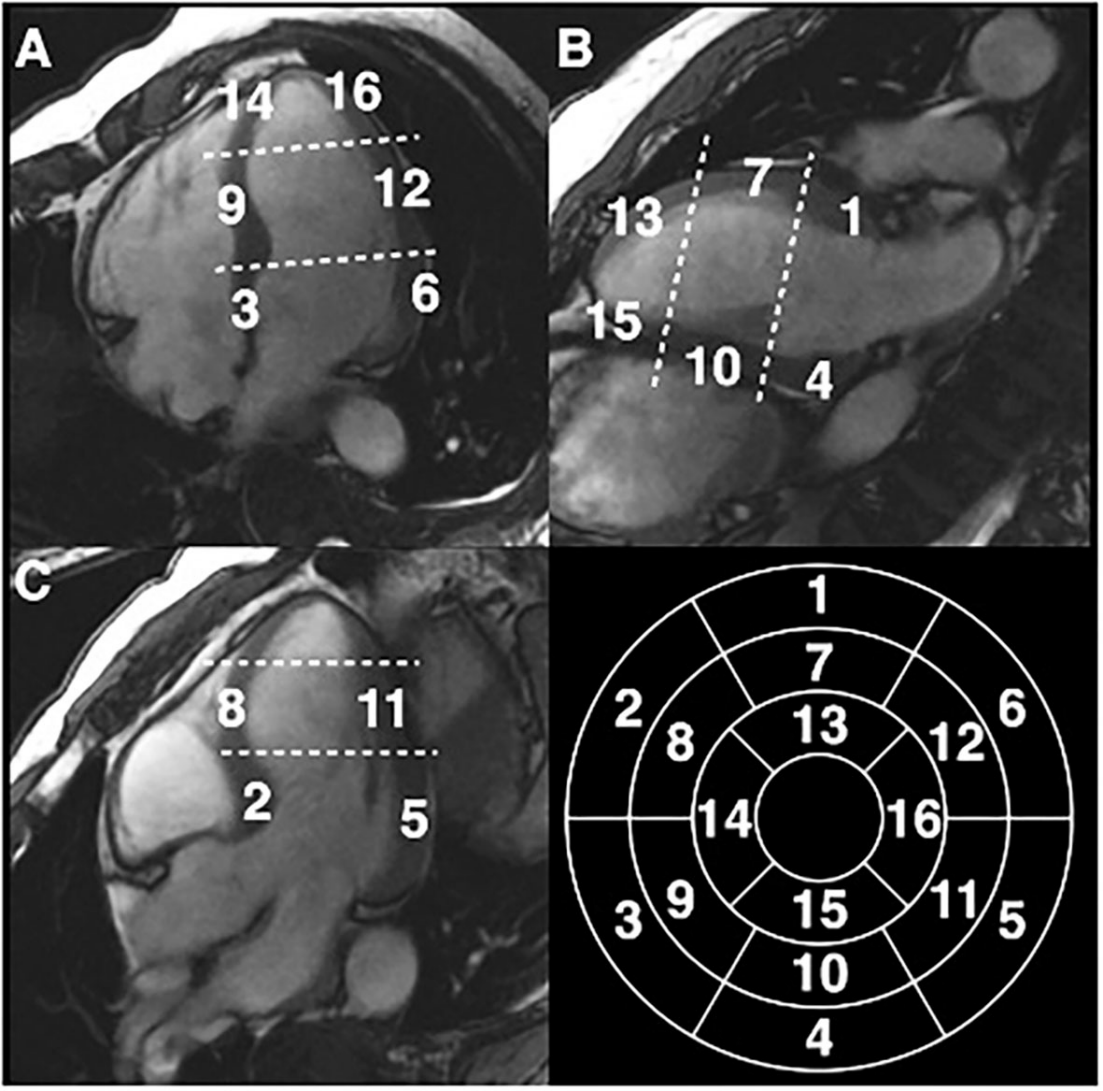
Measurement of NC/C ratios in EuroCMR Registry in 16 segments of the American Heart Association model excluding the true apical cap (segment 17). **(A)** Four chambers view, **(B)** two chambers view, **(C)** three chambers view.

**Figure 3:**
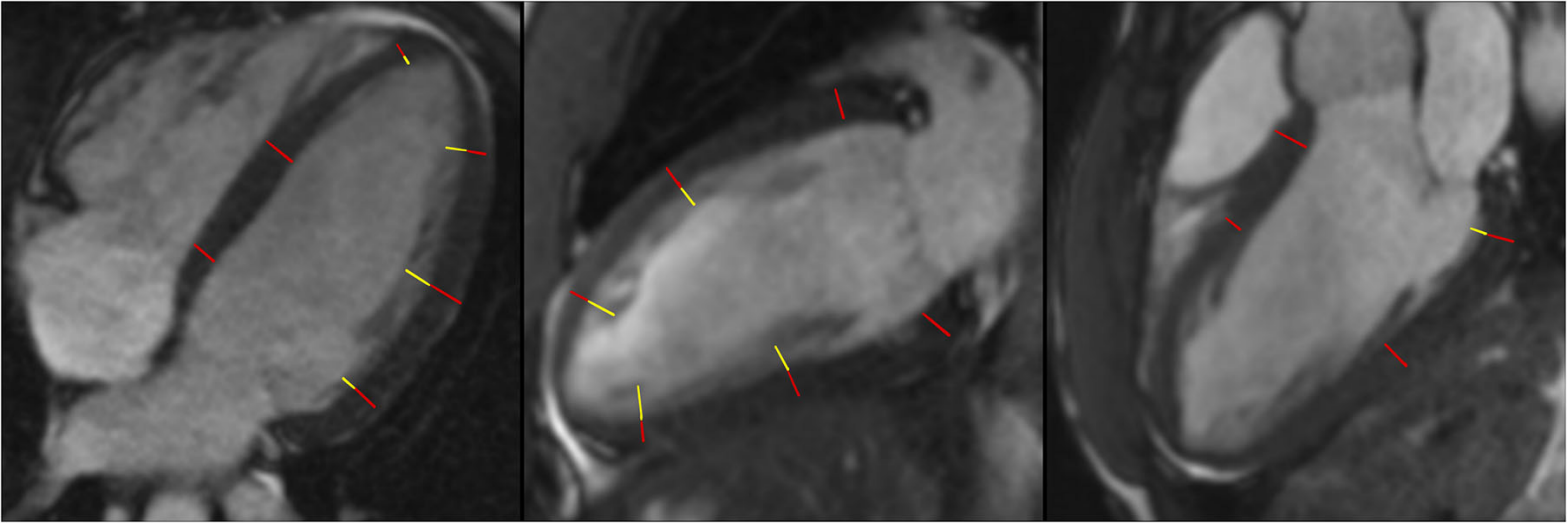
Measurement of NC/C ratios in 4-chamber, 2-chamber, and 3-chamber longitudinal view. Red lines represent compacted myocardium defined as a myocardial layer of homogeneous moderate signal intensity distinctly separate from the blood pool, yellow lines represent measurement of trabeculations defined as a meshwork of moderate signal intensity on the endocardial surface of the compacted myocardium with interspersion of higher SI from the blood pool; NC/C, non-compacted to compacted ratio. SI, signal intensity.

Three long-axis (2-, 3-, and 4-chamber) cine images were used for measuring the thickness of compacted myocardium and trabeculations at the centre of 16 segments of the AHA model (Figure 2). Compacted myocardium was defined as a myocardial layer of homogeneous moderate signal intensity (SI) distinctly separate from the blood pool. Trabeculations were defined as a meshwork of moderate SI on the endocardial surface of the compacted myocardium with interspersion of higher SI from the blood pool (Figure 3). Papillary muscles were excluded from measurements. Short-axis views were used in conjunction with the long axis images to aid identification of papillary muscles. In normal individuals, the true apex is typically thin with prominent trabeculations, it was therefore also excluded from analysis. The maximum ratio of non-compaction to compaction (NC/C) was calculated for each segment. NC/C >2.3 was used as cut-off for LVNC as per the Petersen criteria (8). Measurements of 100 randomly selected studies repeated by the first reader showed excellent intraobserver variability (intraclass correlation coefficients (ICC): LVEDVi 0.98, LVESVi 0.95, LVEF 0.83, LVMi 0.97; *p* < 0.0001 for all parameters). The interobserver variability in the same 100 studies of LV measurements in our group showed very good to excellent reproducibility; ICC values: LVEDVi 0.97, LVESVi 0.88, LVEF 0.71, LVMi 0.92; *p* < 0.0001 for all measurements.

### Outcomes

We considered the following outcomes: all-cause mortality, stroke, atrial fibrillation, severe heart failure (defined as New York Heart Association class 3 and 4). Outcomes were obtained from standardized EuroCMR interviews with follow up duration of 404 ± 82 days. Additionally, we investigated the association of demographic and baseline characteristics on LV trabeculation extent.

### Statistical Analysis

Statistical analysis was performed using R version 3.6.1 (2019-07-05) [R Core Team (2013). R: A language and environment for statistical computing. R Foundation for Statistical Computing, Vienna, Austria. URL: http://www.R-project.org/]. Continuous variables are summarized with mean (standard deviation, SD) or median (interquartile range, IQR). Categorical variables are presented as frequencies and percentages. Differences between LV trabeculation quintiles were evaluated by the analysis of variance (ANOVA) with a *post-hoc* Tukey test for continuous variables and chi-squared test for categorical variables. Univariate linear regression models were used to assess the relationship between demographic factors, clinical data and LV structural parameters as exposure variables and maximal NC/C as the outcome variable. Cox proportional hazards regression models were used to estimate hazard ratios (HR) and the associated 95% confidence interval (CI) for mortality. Univariable logistic-regression models were used to estimate the odds ratio (OR) and the associated 95% CI for the endpoints. LV trabeculation may be more relevant to individuals with suspected non-ischaemic cardiomyopathies. Therefore, subgroup analysis was performed on a subgroup of 569 subjects without ischaemic heart disease (IHD), defined as having no history of myocardial infarction, coronary revascularisation, or evidence of IHD on CMR.

## Results

### Baseline Demographics and Indications for CMR

Demographic data are presented in Table 1. Sixty three percenatge (*n* = 516) of participants were male. Mean age was 59 ± 14 years (range: 16–90). There was substantial burden of cardiovascular risk factors: hypertension 60%, diabetes 14%, dyslipidaemia 40%, smoking history 29%. The most common indications for CMR were evaluation of coronary artery disease (81.9%), cardiomyopathy (15.7%), and myocarditis (8.5%), there was non-exclusivity of indications.

**Table 1:** Summary of baseline demographic and selected cardiac magnetic resonance data.

	**All**	**Quintile 1**	**Quintile 2**	**Quintile 3**	**Quintile 4**	**Quintile 5**	***p*-value**
NC/C		0.55–1.29	1.3–1.54	1.55–1.83	1.84–2.22	2.23–5.64	
*n*	822	166	167	161	166	162	
**Baseline demographics and cardiovascular risk factors**							
Age (years)	59.4 ± 13.9	60.9 ± 13.8	59.7 ± 14.6	59.7 ± 13.9	60.5 ± 13.1	56.1 ± 13.8	<0.05
Females	306 (37.2%)	53 (31.9%)	57 (34.1%)	62 (38.5%)	62 (37.3%)	72 (44.4%)	0.18
Body mass index (kg/m^2^)	27.6 ± 5.9	28.0 ± 5.0	27.9 ± 7.3	26.6 ± 4.0	28.1 ± 6.8	27.1 ± 5.8	0.11
Height (cm)	172.2 ± 9.8	172.6 ± 9.5	172.4 ± 11.1	171.9 ± 9.3	172.1 ± 9.4	171.9 ± 9.8	0.96
Weight (kg)	81.8 ± 17.3	83.5 ± 16.5	82.3 ± 15.5	78.9 ± 14.4	83.4 ± 19.3	80.9 ± 19.8	0.08
Hypertension (*n* = 626)	357 (57.0%)	90 (70.3%)	71 (55.5%)	67 (55.8%)	71 (54.2%)	58 (48.7%)	<0.05
Diabetes (*n* = 626)	86 (13.7%)	21 (16.4%)	17 (13.3%)	17 (14.2%)	24 (18.3%)	7 (5.9%)	0.053
Dyslipidaemia (*n* = 625)	250 (40%)	51 (39.8%)	46 (35.9%)	55 (45.8%)	55 (42.3%)	43 (36.1%)	0.46
Family history of CAD (*n* = 597)	166 (27.8%)	40 (31.7%)	27 (21.8%)	37 (32.5%)	33 (27.3%)	29 (25.9%)	0.32
**Smoking (*****n*** **=** **626)**							
Never	445 (71.1%)	93 (72.7%)	85 (66.4%)	88 (73.3%)	89 (67.9%)	90 (75.6%)	0.46
Former	95 (15.2%)	15 (11.7%)	26 (20.3%)	14 (11.7%)	26 (19.8%)	14 (11.8%)	0.08
Current	86 (13.7%)	20 (15.6%)	17 (13.3%)	18 (15%)	16 (12.2%)	15 (12.6%)	0.92
**Important medical history (*****n*** **=** **809)**							
Myocardial infarction	51 (6.3%)	11 (6.6%)	8 (4.8%)	12 (7.5%)	13 (8.0%)	7 (4.5%)	0.6
CABG	19 (2.3%)	9 (5.4%)	2 (1.2%)	2 (1.3%)	4 (2.5%)	2 (1.3%)	0.052
PCI	78 (9.6%)	13 (7.8%)	18 (10.9%)	18 (11.3%)	20 (12.3%)	9 (5.7%)	0.23
Stroke	28 (3.5%)	6 (3.6%)	4 (2.4%)	10 (6.3%)	6 (3.7%)	2 (1.3%)	0.16
PVD	26 (3.2%)	5 (3.0%)	9 (5.5%)	3 (1.9%)	5 (3.1%)	4 (2.5%)	0.43
Malignancy	30 (3.7%)	5 (3.0%)	5 (3.0%)	7 (4.4%)	7 (4.3%)	6 (3.8%)	0.93
**ECG findings**							
Heart rate (bpm) (*n* = 820)	68.6 ± 13.1	68.6 ± 12.8	68.5 ± 11.9	69.6 ± 12.8	68.6 ± 15.1	67.6 ± 12.5	0.74
Atrial fibrillation (*n* = 822)	20 (2.4%)	6 (3.6%)	2 (1.2%)	4 (2.5%)	5 (3.0%)	3 (1.9%)	0.64
Ventricular extrasystoles (≥ 10/min)	28 (3.4%)	4 (2.4%)	7 (4.2%)	7 (4.3%)	6 (3.6%)	4 (2.5%)	0.79
**CMR parameters**							
LVEDVi (ml/m^2^)	79 ± 25.3	74.5 ± 27.8	75.5 ± 20.7	79.6 ± 24.4	80.3 ± 23.4	85.4 ± 28.2	<0.001
LVESVi (ml/m^2^)	36 ± 22.5	33.9 ± 25.0	32.8 ± 18.0	35.9 ± 20.8	37.4 ± 21.9	40.1 ± 25.6	<0.5
LVEF (%)	56.5 ± 11.6	56.9 ± 11.7	58.3 ± 10.3	56.5 ± 10.7	55.4 ± 13.4	55.2 ± 11.7	0.1
LVEF ≤ 35%	46 (5.6%)	9 (5.4)	5 (3.0)	8 (5.0)	12 (7.2)	12 (7.4)	0.39
LVMi (g/m^2^)	54.7 ± 17	59.0 ± 21.1	55.7 ± 17.0	54.1 ± 16.5	53.0 ± 13.8	51.4 ± 15.0	<0.01

*bpm, beats per minute; CAD, coronary artery disease; EDV, end diastolic volume; EF, ejection fraction; ESV, end systolic volume; g, gram; i, indexed; LVM, LV mass; LV, left ventricle; m, meter; min, minute; NC/C, non-compaction to compaction ratio. Numbers displayed are mean ± standard deviation or absolute number and percentage (please note, the denominator varies depending on data completeness)*.

### Diagnoses Following CMR

No abnormality was detected in 49% of the CMR scans. IHD was diagnosed in 25% of patients. Amongst these, myocardial ischaemia was detected in 147 cases, syndrome X in 64, and cardiac thrombus in one patient. Non-ischaemic cardiomyopathies were diagnosed in 111 patients: HCM (*n* = 59), dilated cardiomyopathy (*n* = 33), arrhythmogenic right ventricular cardiomyopathy (*n* = 1), unclassified cardiomyopathy (*n* = 18). Myocarditis was diagnosed in 29 patients.

### LV Trabeculation Extent

NC/C was calculated in 10,456 (79.5%) segments. The remaining 2,696 segments either had insufficient contrast between the blood pool and the myocardium or were off-axis impeding confident measurement of NC/C. NC/C >2.3 was measured in at least one segment for 17.2% of participants (*n* = 142), and in at least two segments for 5.1% (*n* = 42). The maximal NC/C had a log-normal distribution. The mean maximal NC/C for each participant's analyzed segments was 1.81 ± 0.67. Greater magnitude of trabeculation was detected in lateral segments, and increased from base to apex. LV trabeculation was not seen in the basal infero-septal segment of any participant.

### LV Volumes, Mass, and Systolic Function

LV volumes and functional parameters are presented in Table 1. Severe LV systolic dysfunction (LVEF **≤**35%) was detected in 5.6% (*n* = 46). There were no differences between the quintiles of maximal NC/C in the frequency of severe LV systolic dysfunction (*p* = 0.39).

### Determinants of Maximal NC/C

NC/C was higher in women than in men (1.89 ± 0.72 vs. 1.77 ± 0.64, *p* < 0.05) and decreased with age (β = −0.05 per decade, *p* < 0.01). Maximal NC/C was larger by 0.5 for every 100 ml/m^2^ increase in LVEDVi (*p* < 0.0001), and by 0.4 for every 100 ml/m^2^ increase in LVESVi (*p* < 0.0001). Maximal NC/C was also larger by 0.06 units for every 10% decrease in LV ejection fraction (*p* < 0.01).

### Association of LV Trabeculation With Mortality

Mortality data was available for 530 participants with mean follow-up of 404 ± 82 days. During the follow up period, 10 deaths were recorded (death rate of 1.9%). The extent of maximal NC/C was not associated with increased mortality. There was no difference in survival between quintiles of maximal NC/C (Table 2). Similarly, there was no excess mortality in those with the highest NC/C (quintile 5, NC/C 2.23 to 5.64) compared to the remaining participants (OR 0.51, 95% CI: 0.03–2.76, *p*-value 0.53).

**Table 2:** Predictors of mortality in the EuroCMR Registry.

	**Odds ratio**	***p*-value**	**Hazard ratio**	***p*-value**
**Baseline demographics and cardiovascular risk factors**				
Age per year	1.02 (0.97–1.08)	0.38	1.02 (0.97–1.08)	0.38
Females	1.09 (0.27–3.87)	0.89	1.08 (0.3–3.8)	0.91
Body mass index per kg/m^2^	1.01 (0.89–1.07)	0.85	1.01 (0.92–1.1)	0.83
Height per cm	1.03 (0.96–1.1)	0.42	1.03 (0.96–1.1)	0.43
Weight per kg	1.01 (0.98–1.04)	0.48	1.01 (0.98–1.04)	0.48
Hypertension	0.97 (0.21–5.0)	0.97	0.96 (0.22–4.3)	0.96
Diabetes	1.17 (0.06–7.01)	0.89	1.16 (0.14–9.63)	0.89
Dyslipidaemia	1.09 (0.21–5.01)	0.91	1.1 (0.25–4.9)	0.9
Smoking	1.0 (0.14–4.69)	0.99	1 (0.19–5.15)	1
**CMR volume and function measures**				
LV end-diastolic volume index per ml/m^2^	1.02 (1–1.04)	<0.05	1.02 (1.002–1.038)	<0.05
LV end-systolic volume index per ml/m^2^	1.02 (1.002–1.04)	<0.05	1.02 (1.005–1.036)	<0.01
LV ejection fraction per%	0.94 (0.91–0.98)	<0.01	0.94 (0.91–0.980)	<0.001
LV mass index per g/m^2^	1.03 (1.01–1.05)	<0.01	1.03 (1.01–1.05)	<0.01
Late gadolinium enhancement	6.4 (1.79–25.35)	<0.01	6.1 (1.7–21.8)	<0.01
**Maximal NC/C as a continuous variable**				
Maximal NC/C per 1 unit	1.39 (0.52–2.84)	0.44	1.38 (0.61–3.13)	0.44
**Quintiles of maximal NC/C vs. 1**				
Quintile 2	No events	0.99		1
Quintile 3	2.1 (0.4–15.4)	0.4	2.04 (0.4–11.1)	0.41
Quintile 4	1.59 (0.3–12.2)	0.62	1.6 (0.3–9.4)	0.62
Quintile 5	0.59 (0.03–6.2)	0.67	0.58 (0.05–6.4)	0.66
Quintile 5 vs. remaining participants	0.51 (0.03–2.76)	0.53	0.5 (0.06–4.0)	0.52
Fulfilled Petersen's LVNC criteria (NC/C *C* ≥ 2.3)	0.62 (0.03–3.37)	0.65	0.62 (0.08–4.87)	0.65
**Number of segments with NC/C** **≥** **2.3**				
1	No events	0.99		0.99
2	3.06 (0.16–17.8)	0.3	2.99 (0.38–23.6)	0.3
3	No events	0.99		1
4	No events	0.99		1

*cm, centimeter; g, gram; kg, kilogram; m, meter; ml, milliliter; NC/C, non-compacted to compacted ratio*.

### Association of LV Trabeculation With Clinical Outcomes

#### Heart Failure

Data on heart failure status assessed by the New York Heart Association (NYHA) classification was available for 513 participants. Of these, 65% (*n* = 333) were in NYHA class I, 11.7% (*n* = 60) in NYHA class II, 3.7% (*n* = 19) in NYHA class III, and <1% (*n* = 5) in NYHA class IV. Increasing quintiles of the maximal NC/C were not associated with greater odds of severe heart failure defined as NYHA class III and IV (Table 3). Sensitivity analyses looking at (1) Quintile 5 vs. the remaining cohort, (2) LVNC >2.3, and (3) number of segments with NC/C>2.3 showed similar results.

**Table 3:** Associations of maximal NC/C ratio with severe heart failure, stroke, and MACE.

	**Severe heart failure (NYHA III/IV)**		**Stroke**		**MACE**	
	**Odds ratio**	***p*-value**	**Odds ratio**	***p*-value**	**Odds ratio**	***p*-value**
**Maximal NC/C as a continuous variable**						
Maximal NC/C per 1 unit	0.65 (0.28–1.33)	0.28	0.82 (0.41–1.50)	0.56	0.81 (0.49–1.29)	0.39
**Quintiles of maximal NC/C vs. quintile 1**						
Quintile 2	0.82 (0.2–3.2)	0.78	0.69 (0.17–2.5)	0.58	0.62 (0.24–1.55)	0.32
Quintile 3	1.99 (0.66–6.67)	0.23	1.85 (0.66–5.6)	0.25	1.57 (0.67–2.7)	0.26
Quintile 4	0.64 (0.13–2.69)	0.55	1.11 (0.34–3.7)	0.86	0.89 (0.46–2.0)	0.8
Quintile 5	0.71 (0.14–2.97)	0.64	0.6 (0.12–2.35)	0.48	0.62 (0.27–1.45)	0.33
Quintile 5 vs. remaining participants	0.64 (0.14–1.91)	0.48	0.52 (0.12–1.52)	0.29	0.62 (0.25–1.33)	0.26
Fulfilled Petersen's LVNC criteria (NC/C ≥2.3)	0.49 (0.08–1.72)	0.35	0.41 (0.06–1.4)	0.23	0.52 (0.17–1.22)	0.17
**Number of segments with NC/C** **≥** **2.3**						
1	0.34 (0.02–1.66)	0.3	0.56 (0.09–1.96)	0.45	0.42 (0.1–1.21)	0.16
2	1.25 (0.07–6.63)	0.83	No events	0.99	1.03 (0.16–3.83)	0.97
3	No events	0.99	No events	0.99	No events	0.99
4	No events	0.99	No events	0.99	No events	0.99

*MACE, major adverse cardiac events defined as severe heart failure, stroke, and death; NC/C, non compaction to compaction ratio; NYHA, New York Heart Association*.

#### Stroke

Twenty-eight (3.4%) participants had prior history of stroke. There were no differences in the prevalence of stroke between the quintiles of maximal NC/C (*p* = 0.16) (Table 1). One participant developed a stroke during follow-up. His maximal NC/C was 2.38. Inclusion of this case in the analysis did not alter the results (Table 3).

#### Atrial Fibrillation

Twenty (2.4%) participants had a history of atrial fibrillation. There were no differences in the frequency of atrial fibrillation among quintiles of maximal NC/C (*p* = 0.16) (Table 1).

#### Major Adverse Cardiovascular Events (MACE)

MACE was defined as the composite of all-cause mortality (*n* = 10), severe heart failure (*n* = 24), and stroke (*prevalent* = *28, incident* = *1*). Some participants had more than more of these outcomes, overall, 57 (12.8%) individuals had at least one MACE. The extent of maximal NC/C expressed as a continuous variable and in quintiles was not associated with frequency of MACE (Table 3). Sensitivity analysis did not show associations between other LV trabeculation extent measures and the frequency of MACE.

## Subgroup Analysis of Individuals Without IHD

The 569 individuals without IHD were younger (*p* < 0.01) and more likely to be women (*p* < 0.05). They had lower prevalence of diabetes (*p* < 0.0001) and hypertension (*p* < 0.05) (Table 4). A greater proportion of individuals in this cohort had NC/C >2.3 compared to the whole cohort (19.2 vs. 17.2%). However, the mean maximal NC/C was comparable to the whole cohort at 1.82 ± 0.7. There was greater mortality risk in individuals without IHD, with 8 of the 10 observed deaths occurring in this group. The overall MACE was lower than the whole cohort (*n* = 35, 9.6% vs. *n* = 57, 12.8%). There was no association between maximal NC/C and mortality (OR 1.19, 95% CI: 0.39–2.62, *p* = 0.72) or MACE (OR 0.65, 95% CI: 0.34–1.15, *p* = 0.17). The number of individual outcomes (other than all-cause mortality) was too small in this subgroup for sufficiently powered statistical analysis.

**Table 4:** Comparison of baseline data for the subgroup without IHD with the whole cohort.

	**All (*n* = 822)**	**No IHD (*n* = 569)**	***p*-value**
**Baseline demographics and cardiovascular risk factors**			
Age (years)	59.4 ± 13.9	57.3 ± 14.3	<0.01
Females	306 (37.2%)	224 (39.4%)	<0.05
Body mass index (kg/m^2^)	27.6 ± 5.9	27.7 ± 6.5	0.71
Height (cm)	172.2 ± 9.8	172.3 ± 9.9	0.90
Weight (kg)	81.8 ± 17.3	82.2 ± 18.2	0.75
Hypertension (*n* = 626)	357 (57.0%)	238 (54.1%)	<0.05
Diabetes (*n* = 626)	86 (13.7%)	43 (9.8%)	<0.0001
Dyslipidaemia (*n* = 625)	250 (40%)	159 (36.2%)	<0.01
Family history of coronary disease (*n* = 597)	166 (27.8%)	114 (27.2%)	0.56
**Smoking history (*****n*** **=** **626)**			
Never	445 (71.1%)	313 (71.1%)	0.90
Former	95 (15.2%)	67 (15.2%)	0.99
Current	86 (13.7%)	60 (13.6%)	0.87
**Previous medical history (*****n*** **=** **809)**			
Myocardial infarction	51 (6.3%)	0	<0.0001
CABG	19 (2.3%)	0	<0.0001
PCI	78 (9.6%)	0	<0.0001
Stroke	28 (3.5%)	13 (2.3%)	<0.01
PVD	26 (3.2%)	12 (2.1%)	<0.01
Malignancy	30 (3.7%)	16 (2.8%)	<0.05
**ECG findings**			
Heart rate (bpm) (*n* = 820)	68.6 ± 13.1	68.9 ± 13.3	0.67
Atrial fibrillation (*n*=822)	20 (2.4%)	11 (1.9%)	0.24
Ventricular extrasystoles (≥ 10/min)	28 (3.4%)	21 (3.7%)	0.56
**CMR parameters**			
LV end-diastolic volume index (ml/m^2^)	79.0 ± 25.3	78.3 ± 24.5	0.63
LV end-systolic volume index (ml/m^2^)	36.0 ± 22.5	34.9 ± 21.0	0.37
LV ejection fraction (%)	56.5 ± 11.6	57.3 ± 11.1	0.19
Severe LVSD (EF <35%)	46 (5.6%)	29 (5.1%)	0.54
LV mass index (g/m^2^)	54.7 ± 17	53.8 ± 17.2	0.37

*Bpm, beats per minute; CABG, coronary artery bypass graft; cm, centimeter; g, gram; IHD, ischaemic heart disease; kg, kilogram; LV, left ventricle; m, meter; ml, milliliter; PCI, percutaneous coronary intervention; PVD, peripheral vascular disease. Numbers displayed are mean ± standard deviation or absolute number and percentage (please note, the denominator varies depending on data completeness)*.

## Discussion

### Summary of Findings

In this multicenter multinational study of real-life patients with clinical indication for CMR, we identified no association between the extent of LV trabeculation and increased risk of all-cause mortality, severe heart failure, stroke, atrial fibrillation, or MACE composed of all these parameters. The same result was observed with subgroup analysis of individuals without IHD and with stratified analysis using the threshold of NC/C >2.3. Women and younger individuals had greater magnitudes of LV trabeculation, as did those with larger cavity volumes and lower LVEF.

### Strengths and Limitations

Whilst the inclusion of consecutive real-life clinical patients is a strength of this study, analysis of individuals with relevant symptoms in a more selective manner may provide more meaningful context for interpretation of clinical significance of LV trabeculation. However, this approach would introduce ascertainment bias, which has hampered previous studies with highly selective cohorts. We only used one method to quantify trabeculation extent. However, whilst other methods exist (22), method of measurement does not appear to result in important differences (23). Substantial number of participants had missing data for the mortality outcome (*n* = 292), we limited our analysis to individuals with an explicitly documented mortality outcome (dead/alive). Of course, there is still potential for bias, e.g., relating to excess deaths in the missing cohort, however, we believe our approach is the method with least potential for bias within the limitations of the information available. Our observations reflect short-term risk with an average follow-up period of just over 12 months; we cannot exclude longer term prognostic significance of LV trabeculation.

### Comparison With Existing Literature

The proportion of individuals in our study with NC/C >2.3 in at least one segment was high (17%, *n* = 142), however this did not translate to an exaggerated rate of adverse events. Similar prevalence of LV hyper-trabeculation has been reported in multiple studies of healthy cohorts with no association to poor outcomes (9, 11). Notably, analysis of 2,742 asymptomatic individuals free of cardiovascular disease from the multi-ethnic study of atherosclerosis (MESA) with 9.5 years follow-up showed no association between adverse cardiac remodeling and LV trabeculation extent (10). Amzulescu et al. report more frequent observation of LV hyper-trabeculation in a cohort of 162 dilated cardiomyopathy patients (NC/C ≥ 2.3 in 36%). However, clinical outcomes were related to left and right ventricular remodeling and the presence of late gadolinium enhancement (LGE), and not trabeculation extent (16). High rates of excess LV trabeculation were also reported in a study of 101 healthy pregnant women (25.4%), with resolution in 73% after childbirth (12). Studies of healthy athletes also report higher rates of excess LV trabeculation with no clear prognostic relevance (13). These cohorts have in common LV dilatation as part of their LV remodeling phenotype. It is conceivable, that increased cavity size allows better visualization and delineation of trabeculae, thereby introducing a systematic measurement error toward over-estimation. Indeed, we also observed greater measures of LV trabeculation in individuals with larger LV volumes. Systematic over-estimation of the trabecular component seems more likely than genuine increased trabeculation in these populations or *de-novo* appearance and spontaneous disappearance of LV trabeculae in pregnancy and post-partum as had been suggested (12).

Ivanov et al. present the only other study, aside from ours, assessing the significance of LV trabeculation extent in a real-life clinical setting (15). Consistent with our findings, they report no association between LV trabeculation extent and adverse clinical events (death, ischemic stroke, ventricular tachycardia/ventricular fibrillation, heart failure hospitalization) in 700 consecutive patients undergoing clinical CMR in a single centre over 7 years of prospective follow-up. Interestingly, a multicenter Italian study with a more selective population of individuals with a diagnosis of LVNC based on clinical and imaging criteria also reports no prognostic value of LV trabeculation extent above and beyond LV dilation, LV systolic dysfunction, and presence of LGE over 2 years of prospective follow-up (17).

Thus, there is accumulating evidence from multiple clinical studies, with a variety of study designs, in different populations disputing the clinical significance of LV hyper-trabeculation. Furthermore, the genetic and embryologic origins of LVNC have been questioned. Although several genetic links to LVNC have been established, there is significant overlap with other cardiomyopathies and no single gene has been reliably and specifically linked to LVNC (24). Additionally, the embryologic origins of LVNC have been disputed with recent studies contesting the existence of an *in-utero* “myocardial compaction” phase (24–26). The intra-uterine period is a hugely important phase of cardiac development, during which the cardiovascular system is exposed to marked haemodynamic change and detectable changes in adult cardiac morphology have been demonstrated with alterations in the intra-uterine environment (27). It is conceivable that the observed differences in patterns of LV trabeculation are an epiphenomenon reflecting the haemodynamic changes, rates of myocardial growth, and myocardial differentiation *in-utero* rather than a distinct genetic cardiomyopathy.

Whilst initially presented as a rare genetic cardiomyopathy with poor outcomes, growing evidence from a number of sources in different populations shows high prevalence of LV hyper-trabeculation by imaging criteria with no evidence of associated prognostic significance. Furthermore, the genetic and embryologic evidence for the etiology of LVNC are equivocal, with the absence of a clear gene-phenotype link and lack of evidence for an embryologic myocardial compaction phase. It seems increasingly likely that the initial reported poor outcomes relating to LVNC were the biased results of highly selective tertiary centre cohorts. It is clear from the available evidence, that the current imaging criteria for quantification of LV trabeculation are too broad, capturing high proportions of individuals with no underlying cardiac disease and no increased risk of adverse events.

## Conclusion

This study adds to the growing evidence that LV hyper-trabeculation in isolation is not a predictor of risk and does not mandate further investigation or follow-up. In cases, where there is uncertainty regarding the existence of LVNC as a distinct cardiomyopathy, physicians should take a holistic approach and exert caution in making this diagnosis.

## Data Availability Statement

The raw data supporting the conclusions of this article will be made available by the authors, without undue reservation.

## Ethics Statement

All participating center had approval from local institutional ethics review boards, and all patients provided written informed consent in accordance to the Declaration of Helsinki, under standards of the EuroCMR registry. The patients/participants provided their written informed consent to participate in this study.

## Author Contributions

FZ, SEP, and SM conceived the study. FZ collated, analyzed the CMR images, and led and performed the statistical analysis. ZR-E, FZ, and SEP wrote the manuscript. ZR-E contributed to cross-checking of the analysis. MK contributed to image analysis. All co-authors contributed to critical revision of the manuscript and approved the final version.

## Conflict of Interest

The authors declare that the research was conducted in the absence of any commercial or financial relationships that could be construed as a potential conflict of interest.
