# Supplementary material for: Left Ventricular Hypertrabeculation Is Not Associated With Cardiovascular Morbity or Mortality: Insights From the Eurocmr Registry
Source: Front Cardiovasc Med. 2020 Sep 22;7:158. doi: 10.3389/fcvm.2020.00158 (PMC7536335; doi:10.3389/fcvm.2020.00158)
Supplement: Supplementary file 1 [file Table_1.DOCX]

**Supplementary Table 1. Summary of the EuroCMR registry follow-up interview template**

| **Fatal events** |
| --- |
| Date of death |
| Primary cause of death |
| **Non-fatal events** |
| Aborted sudden cardiac death |
| ICD shocks (Number appropriate/inappropriate/indeterminate) |
| Non-fatal MI |
| Non-fatal stroke |
| Syncope |
| NYHA heart failure class (none, I, II, III, IV) |
| CCS angina grade |
| **Suspected CAD protocol** |
| PCI (Yes/No) |
| CABG (Yes/No) |
| **HCM SCD protocol** |
| Implantation of ICD (Yes/No)  If yes, indication (primary/secondary prevention) |
| Pacemaker placement for outflow tract obstruction |
| Myectomy |
| Alcohol septal ablation |

CABG: coronary artery bypass grafting; CAD: coronary artery disease; CCS: Canadian Cardiovascular Society; HCM: hypertrophic cardiomyopathy; ICD: implantable cardiac defibrillator; MI: myocardial infarction; NYHA- New York Heart Association; PCI: percutaneous coronary intervention; SCD: sudden cardiac death.
